# Electrochemically Active Biofilms as an Indicator of Soil Health

**DOI:** 10.1149/1945-7111/ac1e56

**Published:** 2021-08-26

**Authors:** Abdelrhman Mohamed, Eduardo Sanchez, Natalie Sanchez, Maren L. Friesen, Haluk Beyenal

**Affiliations:** 1 The Gene and Linda Voiland School of Chemical Engineering and Bioengineering, Washington State University, Pullman, Washington, United States of America; 2 Department of Plant Pathology, Washington State University, Pullman, Washington, United States of America; 3 Department of Crop and Soil Sciences, Washington State University, Pullman, Washington, United States of America

## Abstract

Soil health is a complex phenomenon that reflects the ability of soil to support both plant growth and other ecosystem functions. To our knowledge, research on extracellular electron transfer processes in soil environments is limited and could provide novel knowledge and new ways of monitoring soil health. Electrochemical activities in the soil can be studied by inserting inert electrodes. Once the electrode is polarized to a favorable potential, nearby microorganisms attach to the electrodes and grow as biofilms. Biofilms are a major part of the soil and play critical roles in microbial activity and community dynamics. Our work aims to investigate the electrochemical behavior of healthy and unhealthy soils using chronoamperometry and cyclic voltammetry. We developed a bioelectrochemical soil reactor for electrochemical measurements using healthy and unhealthy soils taken from the Cook Agronomy Farm Long-Term Agroecological Research site; the soils showed similar physical and chemical characteristics, but there was higher plant growth where the healthy soil was taken. Using carbon cloth electrodes installed in these soil reactors, we explored the electrochemical signals in these two soils. First, we measured redox variations by depth and found that reducing conditions were prevalent in healthy soils. Current measurements showed distinct differences between healthy and unhealthy soils. Scanning electron microscopy images showed the presence of microbes attached to the electrode for healthy soil but not for unhealthy soil. Glucose addition stimulated current in both soil types and caused differences in cyclic voltammograms between the two soil types to converge. Our work demonstrates that we can use current as a proxy for microbial metabolic activity to distinguish healthy and unhealthy soil.

Soil health—a complex phenomenon that reflects the ability of soil to support both plant growth and other ecosystem functions^1–4^—is fundamentally an emergent property of the microbiomes that live belowground, fueled by resources exuded from plant roots and by decomposition of organic matter in soil systems. The soil and rhizosphere microbiome consist of millions of bacteria, fungi, and other organisms that play critical roles in nutrient mobilization and provisioning, defense against pathogens, and modulation of plant morphology and physiology.^5–15^ Soil health both influences and is influenced by microbial activity and the complex microbial interactions that occur in soil microbiomes. As such, electrochemical methods to monitor microbial activity in the soil could be developed to monitor soil health through direct or indirect measurements of soil microbial activity.

Biofilms are a major part of the soil microbiome and play critical roles in microbial activity and community dynamics. Biofilms in soil are composed of multi-species microbial consortia attached to soil particles and other surfaces, including roots, fungal hyphae, and decomposing organic material.^16^ In 2019, Flemming and Wuertz analyzed the global biofilm abundance and estimated that of the 3 × 10^29^ bacteria and archaea present in the soil, 40%–80% of cells in the subsurface reside in biofilms.^17^ The activity of soil biofilms controls soil structure and physicochemical characteristics, influences water retention and flow, and controls the local chemical gradients in the soil—including nutrients, oxygen, redox potential and pH.^17^ Thus, biofilms drive all biogeochemical processes and represent the main way of bacterial and archaeal life.^17^ When bacteria grow as biofilms in soil, they generate extracellular polymeric substances (EPS) which can be used to support their resilience, electron transfer and soil stability.^18,19^

While research monitoring electrochemical activities in soils is limited, previous work has demonstrated that both abiotic and biotic components of soil likely contribute to overall electrochemical signals. Dissolved organic matter (DOM) represents one of the most mobile and reactive organic compounds in ecosystem and plays an important role in the fate and transport of soil organic content and nutrient cycling.^20,21^ However, some compounds in DOMs are redox-active as found by Yuan et al. (2011), who demonstrated the electron transfer capability of dissolved organic matter (DOM) in soil using cyclic voltammetry (CV) and chronoamperometry (CA).^20^ Following this, Bi et al. (2013) used differential pulse voltammetry (DPV) and CV in combination with spectroscopic techniques (FTIR, UV–vis and fluorescence spectroscopy) to determine the electrochemical and redox properties of DOM in soil.^21^ The microbes growing in soil use electron donors and acceptors in soluble or mineral form for their metabolic reactions and growth. However, solid electrodes polarized at a suitable potential can replace electron donors and acceptors to support microbial growth.^22^ Therefore, it is possible to monitor microbial metabolic activities simply by monitoring the current (electron transfer rate) passing through an electrode with bacteria growing on it. One of the earlier studies in this area showed that polarized electrodes can be used for in situ detection of microbial life in soils.^23^ Following this, Figueredo et al., (2015) demonstrated that polarized electrodes can be used to monitor photosynthetic metabolism.^24^ Similarly, our research group demonstrated how to remotely monitor microbial activities in remote areas using custom-made electronics.^25,26^ In addition to electrochemical measurements, in a recent publication, researchers demonstrated the extracellular electron transfer ability of microbes growing in soil and isolated an electrochemically active bacterial strain CL-1, related to *Geobacter sulfurreducens* subsp.^27^ Since the discovery of electron transfer in soil, researchers have focused on how to increase the rate of this process. For instance, it was claimed that pyrogenic carbon or other conductive carbon-based materials in soil transfer electrons in soil systems.^28^ The addition of pyrogenic carbon is expected to improve soil quality, but this relationship remains to be tested. Recently, polarized electrodes were used to monitor microbial metabolism as current and redox changes in soils.^29^ The addition of electron donors to soil resulted in enhanced biologically produced current, allowing stimulation and detection of dormant microbes.^29^ It was concluded that polarized electrodes in soil provide an approach to detect metabolism in samples without prior knowledge of the microorganisms present and that thorough electrochemical analysis and rigorous experimental design are necessary to determine if signals are due biotic reactions.^29^ Work to date thus demonstrates that soil is a redox active electrochemical system and it is possible to monitor microbial activities using electrochemical techniques. However, research on electron transfer processes in soil environments remains at an early stage, and advances could provide novel knowledge as well as new ways of monitoring soil health.

The goal of our work is to investigate the electrochemical behavior of similar soils that differ in their ability to support plant growth (“healthy” vs “unhealthy”) using open circuit potential (OCP), CA and CV measurements. Soils were collected from similar sites used for wheat production at the Cook Agronomy Farm Long-Term Agroecological Research facility. In this work, soils were designated as “healthy” or “unhealthy” based on historic data for wheat grain yield. We developed a bioelectrochemical soil reactor for electrochemical measurements to study the electrochemical activity of healthy and unhealthy soils. First, we measured redox variations by measuring OCP at different depths in healthy and unhealthy soils. Then, we selected the optimum depth to monitor the electrode current during CA experiments. We used CV before and after CA experiments to understand the change of redox behavior due to electrode polarization. We amended healthy and unhealthy soils with glucose to test if nutrient addition stimulates the metabolic activity and the observed electrochemical signals in healthy and unhealthy soils. Lastly, we harvested the electrodes and used electron microscopy to image cells attached on the electrodes deployed in healthy and unhealthy soils. Our work addresses whether soil electrochemical signals vary between healthy and unhealthy soils and whether microbial metabolism and interactions with electrodes are related to electrochemical signals.

## Materials and Methods

### Soil collection site and soil health.—

We sampled soils from the R. J. Cook Agronomy Farm, a USDA Long Term Agroecosystem Research (LTAR) site providing research data applicable over ∼1 million ha in WA and 405,000 ha in Idaho. The Cook Farm has 615 geo-referenced locations over 57 ha and has been intensively sampled for soil, crop, and terrain properties and an environmental sensor network for modeling biophysical processes (C, N, water) and economic performance over time. Within-field soil pH ranges from 4 to over 7 while soil organic matter ranges from 1 to 5% and is highly stratified with depth. We selected two points on the no-till side of the farm that occurred on the same soil type but that historically showed differences in wheat yield and soil organic matter and collected soil to a depth of 10 cm on September 2 and October 9, 2020 with a hand shovel, following COVID-19 protocols for fieldwork. Note that there was no precipitation between these sampling dates and soil was taken from the same locations, so we are treating these as single samples. Buckets were stored sealed and dry at room temperature.

We obtained historical data from the Cook Agronomy Farm LTAR group on relative wheat grain yield at our two sampling points. For each year, the yield at a point is normalized to the mean yield of the entire site; relative yields were averaged from 1999 to 2015. The “healthy” soil has a mean relative yield of 1.069 and the “unhealthy” soil has a mean relative yield of 0.963.

The LTAR group also obtained soil properties as measured at multiple depths in 2015; however, our “healthy” point was not included in these measurements so we averaged the LTAR data for the two nearest points for the 0–10 cm depth as this is where our soil was collected. These results are shown in SI Table SI (available online at stacks.iop.org/JES/168/087511/mmedia). Three replicate sieved soil subsamples from our soil samples were analyzed by Best Test labs (Moses Lake, WA). This yielded data on: bulk density, electroconductivity, organic matter, NH_4_, NO_3_, P (both Bray and Olsen methods), K (Olsen method), SO_4_, Cl, B, Zn, Mn, Cu, Fe, Na, K, Ca, Mg, Al (KCl and DTPA method), pH, total bases, base saturation, effervescence, estimated cation exchange capacity (CEC), and Ca/CEC, Mg/CEC, Na/CEC, K/CEC. These results are shown in SI Table SII.

### Construction of the electrodes and soil reactors.—

**Figure 1.**
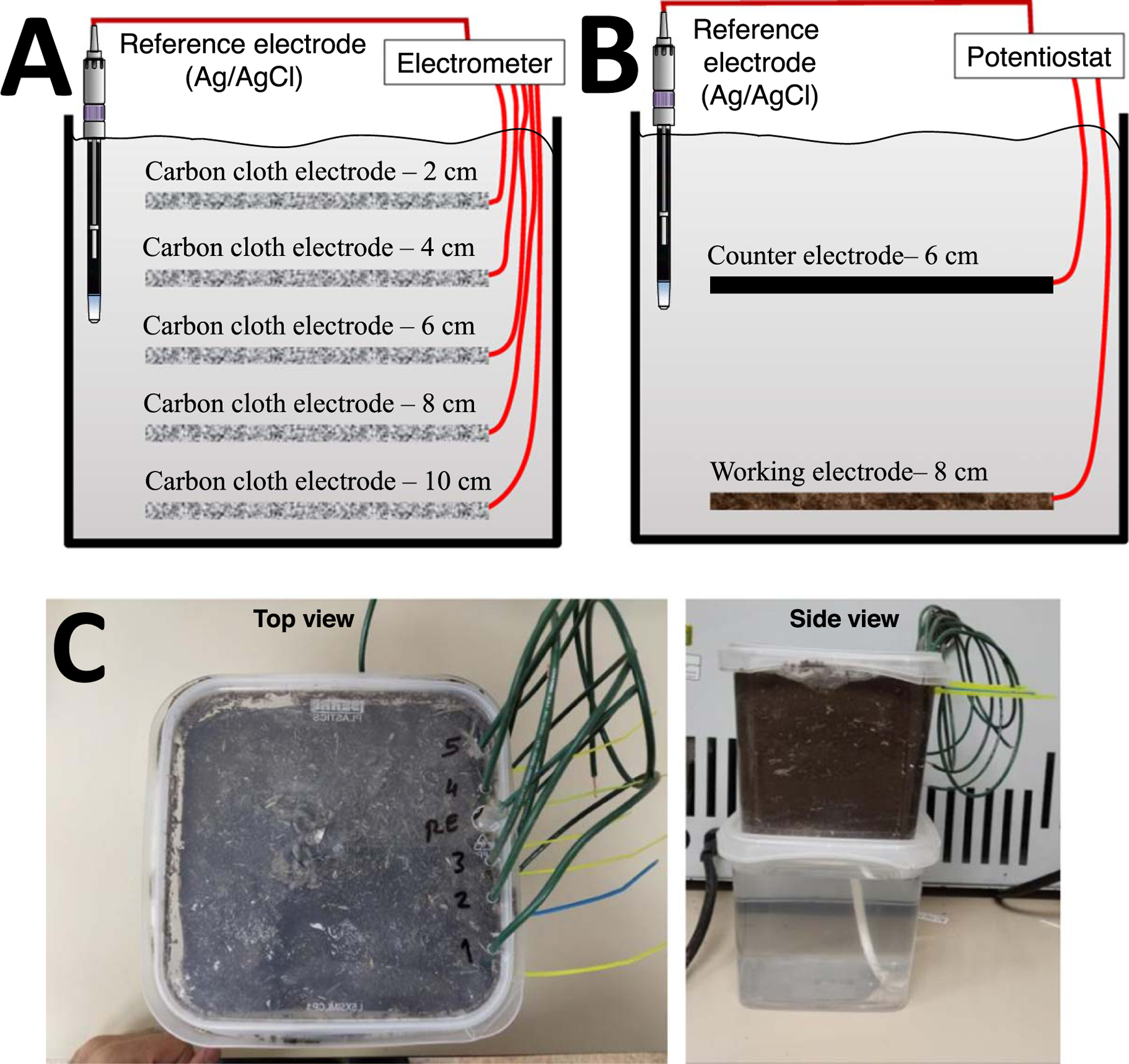
Schematic diagram of the soil reactors used for (A) open circuit potential and (B) biofilm enrichment experiments. (C) A photograph of the soil reactors and the water reservoir. By using a water wick (shown in the side view), we kept water saturation constant in our reactor.

The soil reactors for OCP experiments and for biofilm enrichment experiments are shown in Fig. 1A. First, we constructed reactors to investigate the effect of deployment depth within the soil reactor on the OCP. These reactors consisted of five identical carbon fabric deployed at 2 cm, 4 cm, 6 cm, 8 cm, and 10 cm below the soil surface and a single reference electrode with a porous tip located at 6 cm depth below the soil surface. Similarly, the reactors used for potentiostatic enrichment of electrochemically active biofilms (EABs) consisted of a reference electrode and two identical carbon fabric electrodes: a working electrode and a counter electrode deployed at 8 cm and 6 cm below the soil surface, respectively (Fig. 1B). The reference electrode was placed such that the porous frit is located at a depth between the working and counter electrodes. Carbon fabric electrodes were made of 3.8 cm × 3.8 cm carbon fabric (Zoltek Companies Inc., St. Louis, MO, catalog #PX30FBPW06). Electrical connection to the carbon fabric electrodes was established using a titanium wire woven through the fabric (Malin Co., Cleveland, OH, 0.025-inch diameter, catalog #31262). The titanium wire was wrapped around a plastic screw to ensure a good electrical connection with the carbon electrode; the titanium wire and carbon cloth were placed between the washer and nut then tightened by hand. Two contact points were used for each electrode to ensure the redundancy of the electrical connection. The titanium wires were soldered to an insulated 18 AWG copper wire. The soldering joints were sealed using a marine adhesive sealant (3 M, 5200 Fast Cure, catalog #06535). The maximum resistance between the copper wire and any point on the carbon fabric was 1 Ω or less; otherwise, the electrode assembly was discarded. The reference electrodes were constructed in-house according to previously published protocols.^30^

The soil reactors were assembled in cubic plastic containers shown in Fig. 1C (11 cm × 11 cm × 11 cm) (Berry Plastic 48 oz. PP container part #T5X548IMLCP, with clear lids). Dry soil was sieved with a 1 mm metal sieve to remove debris (including weed seeds) and added to the containers gradually; the carbon electrodes were placed horizontally on the top of the soil at the appropriate depth. The copper wires are connected fixed to the side of the reactor using cable ties to minimize changes in the electrode location during reactor construction. Similarly, the reference electrode was fixed to the side of the reactor using a cable tie and glued using a five-minute epoxy adhesive to prevent movement in the vertical direction (Fig. 1C). The total depth of the soil in the reactors was 11 cm. A braided polypropylene (Fig. 1C—side view) 0.25 inch diameter wick cord was passed through the center of the soil reactors to facilitate water transfer into the soil (ACE Hardware part #75755). The wick cord was extended through a hole at the bottom of the soil containers and inserted into an identical plastic container filled with deionized (DI) water (Fig. 1C—side view). This water reservoir ensured the soil was continuously hydrated throughout the duration of the experiment. In order to initially hydrate the dry soil, the reactors were immersed in a larger container filled with DI to the same height as the soil. Water pressure forced DI water to pass through the hole at the bottom of the soil reactor, through the wick cord and into the dry soil. The soil reactors were removed from the water reservoir once the soil was completely hydrated. All experiments were performed inside an incubator with a controlled temperature at 23 °C. DI water was periodically added to the water reservoir to compensate for water loss.

### Open circuit potential experiments.—

OCP experiments were performed to investigate the effect of deployment depth on the OCP in soil reactors (Fig. 1A). OCP measurements were recorded daily for each electrode using a digital voltmeter (Fluke 87-V, Fluke Corporation, Everett, WA). OCP measurements were recorded for a minimum of 18 days and continued until stable OCP values are established. Data are reported as means and standard deviations of four biological replicates.

### The enrichment of electrochemically active biofilms under constant polarization.—

The electrodes deployed in the soil reactors were polarized at 0.3 V_Ag/AgCl_ to target the enrichment of anodic biofilms on the working electrodes (Fig. 1B). Chronoamperometric measurements were used to monitor the current resulting from biofilm enrichment. An in-house custom potentiostat was used to control the working electrode potential during enrichment and measure the resulting current.^31^ Electrodes were continuously polarized until the current reached a pseudo-steady state (less than 5% change in a day). After reaching a pseudo-steady state current, we amended the soil reactors with a glucose solution to test whether the activity of the biofilms enriched in healthy and unhealthy soils increases in response to the addition of organic carbon sources. Glucose was injected into the soil reactor in the vicinity of the working electrode using a long needle. The exact location could not be determined due to the lack of visibility inside the soil reactor; depth was estimated using length marks made along the needle. A 15 mL volume of 1.85 M glucose was injected; the equivalent of 5 g of glucose was added to each reactor. Chronoamperometric measurements were recorded for 25 days to monitor the response to glucose amendment.

### Electrochemical methods.—

A Gamry 1000^™^ potentiostat was used to record OCP and cyclic voltammetry measurements (Gamry Instruments, Warminster, PA). The measurements were recorded at different time points to monitor biofilm enrichment on the electrochemical activity observed on the working electrode. OCP and cyclic voltammograms were recorded at: 1) day 0—immediately after assembling the soil reactors, 2) day 8—after the initial biofilm enrichment and prior to the addition of glucose, and 3) day 25 and day 36—at two time points after soil amendment with glucose. Cyclic voltammograms were recorded from 0.6 V_Ag/AgCl_ to −0.7 V_Ag/AgCl_ and then back to 0.6 V_Ag/AgCl_ at a scan rate of 0.010 V s^−1^. Three cycles were recorded for each experimental condition. In general, the 2^nd^ and 3^rd^ cycles showed identical responses, which was slightly different from the 1^st^ cycle due to the initial contribution of non-Faradaic currents. The 2^nd^ cycles are reported as representative voltammograms describing the behavior of the biofilm electrodes.

### Scanning electron microscopy.—

Scanning electron microscopy (SEM) was used to provide insight into the enrichment of EABs on polarized electrodes deployed at 8 cm depth in healthy and unhealthy soil reactors. The electrodes were removed from the reactor and immersed overnight in 2% paraformaldehyde, 2% glutaraldehyde in 0.1 M phosphate buffer for primary fixation. The electrodes were then fixed using hexamethyldisilazane, then placed in 2% osmium tetroxide at room temperature for 1 hour and dehydrated immediately. The dehydration process was done using ethanol solutions of 30%, 50%, 70%, 90%, and 100% (10 min per step). After the dehydration, the samples were allowed to dry and then sputter-coated with gold. The electrodes were then placed on aluminum stubs and analyzed by field emission scanning electron microscopy (Tescan Vega3 SEM). Representative images are included for polarized electrodes deployed in healthy and unhealthy soil reactors.

## Results and Discussion

### The effect of deployment depth on open circuit potential.—

**Figure 2.**
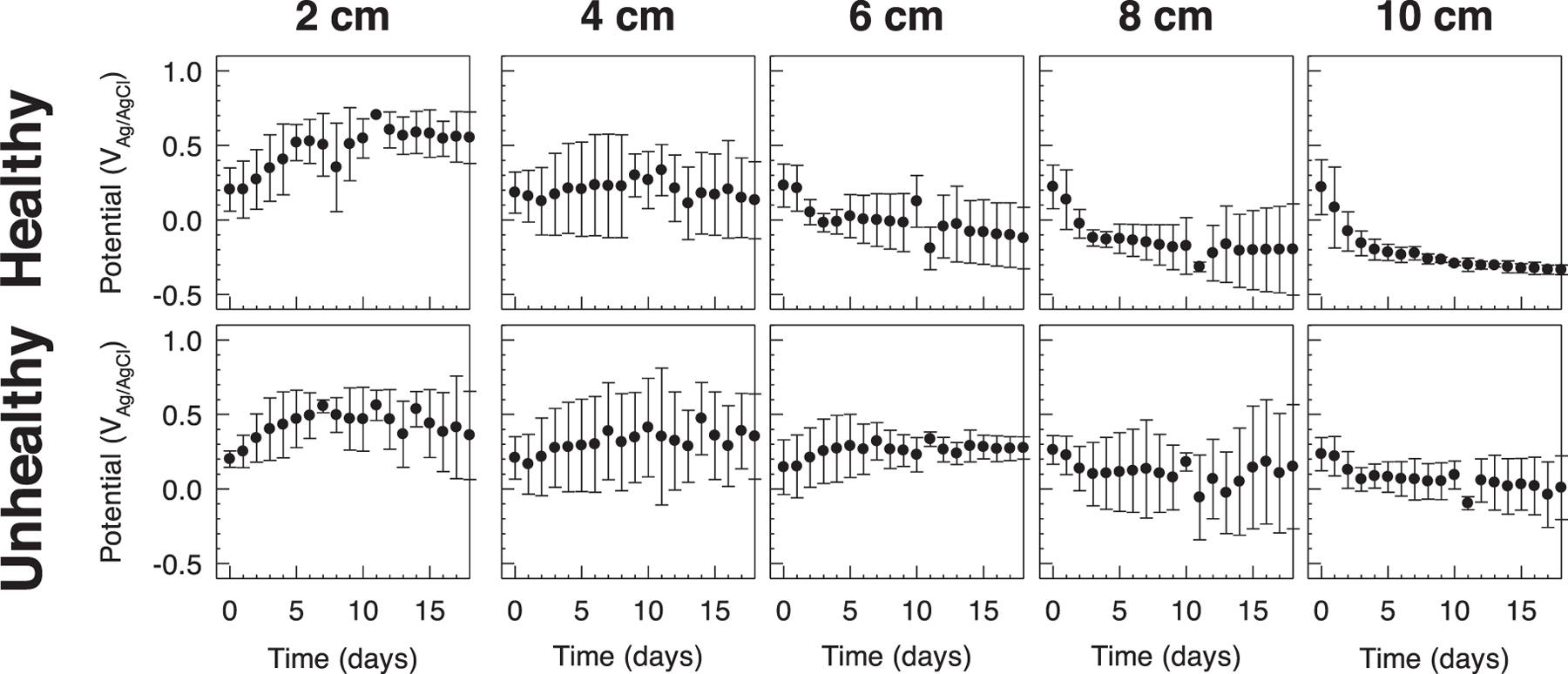
The dependency of deployment depth on the open circuit potential of electrodes deployed in healthy and unhealthy soil reactors. Electrodes were deployed at 2 cm, 4 cm, 6 cm, 8 cm, and 10 cm below the soil surface. Data are represented as means and standard deviations of four biological replicates.

OCP is determined by the redox activity on the surface of the electrode and provides insight into whether the electrode is placed in an oxidizing or a reducing environment. Initially, electrodes deployed in all depths in healthy and unhealthy soils showed a similar OCP range: 0.184 to 0.222 V_Ag/AgCl_ and 0.143 to 0.261 V_Ag/AgCl_ for healthy and unhealthy soils, respectively (Fig. 2). Electrodes near the soil surface (depth = 2 cm) gradually increased their OCP over time in healthy and unhealthy soil reactors. After 18 days, the OCP reached 0.552 ± 0.172 V_Ag/AgCl_ and 0.360 ± 0.296 V_Ag/AgCl_ for healthy and unhealthy soil reactors, respectively. The increase in OCP value indicates that there is a predominantly oxidizing environment, likely due to the establishment of aerobic zone at the top of the soils. The measured OCP values gradually decreased with increasing deployment depth in the healthy soil reactors (Fig. 2). After 18 days, OCP values reached values of 0.133 ± 0.258 V_Ag/AgCl_, −0.122 ± 0.206 V_Ag/AgCl_, −0.197 ± 0.306 V_Ag/AgCl_ and −0.335 ± 0.033 V_Ag/AgCl_ for electrodes deployed at 4 cm, 6 cm, 8 cm and 10 cm in the healthy soil reactors, respectively. It is notable that OCP variability between replicates decreased at the lowest depth (10 cm), indicating that reducing conditions dominated the vicinity of the electrodes. The low OCP values could also indicate that microorganisms utilize the electrode surface as an electron sink if alternative electron acceptors are not readily available. Oxygen could be consumed due to the microbial activity in the soil, causing a gradient of oxygen concentration with depth and the establishment of an anaerobic zone at the deeper parts of the soil. In comparison, a mild decrease in OCP is observed with decreasing deployment depth in the unhealthy soil reactors. The OCP values reached 0.353 ± 0.286 V_Ag/AgCl_, 0.275 ± 0.075 V_Ag/AgCl_, 0.149 ± 0.417 V_Ag/AgCl_ and 0.008 ± 0.213 V_Ag/AgCl_ for electrodes deployed at 4 cm, 6 cm, 8 cm and 10 cm in the unhealthy soil reactors, respectively, after 18 days. Put together, the OCP measurements suggest a higher microbial activity in the healthy soil reactors, causing a larger decrease in measured OCP values which increased with depth. The data indicate that OCP depth gradients could be used as a proxy for microbial activity in soils, and to differentiate between healthy and unhealthy soils. Because reducing environments are prevalent in the deeper parts of healthy soil reactors, we hypothesize that such an environment provides more favorable conditions for the enrichment of anodic EABs which can utilize electrodes polarized at oxidizing potentials as an electron sink. Successful enrichment of anodic EABs could be used as an indicator for metabolic activity near the vicinity of the electrode surface, which could be developed as a tool to infer soil health.

### Differential response of polarized electrodes deployed in healthy and unhealthy soil reactors.—

OCP data shown in Fig. 2 indicate that a reducing environment is dominant in depths below 6 cm. The reducing environment was more evident in healthy soil in comparison to unhealthy soil reactors, suggesting the measured OCP is influenced by microbial metabolism due to oxygen consumption in the top layers of the soil or due to extracellular electron transfer to the electrodes. The availability of microbes capable of extracellular electron transfer could be utilized as a working principle of an electrochemical sensor to monitor soil health. The enrichment of anodic EABs on the surface of polarized electrodes allows monitoring electrode current as a proxy for the level of microbial metabolism in the vicinity of the electrode surface. Since healthy soil can support the metabolism of soil microbes at a higher rate compared to unhealthy soils, we expect a higher current to be observed in electrodes deployed in healthy soil reactors.

**Figure 3.**
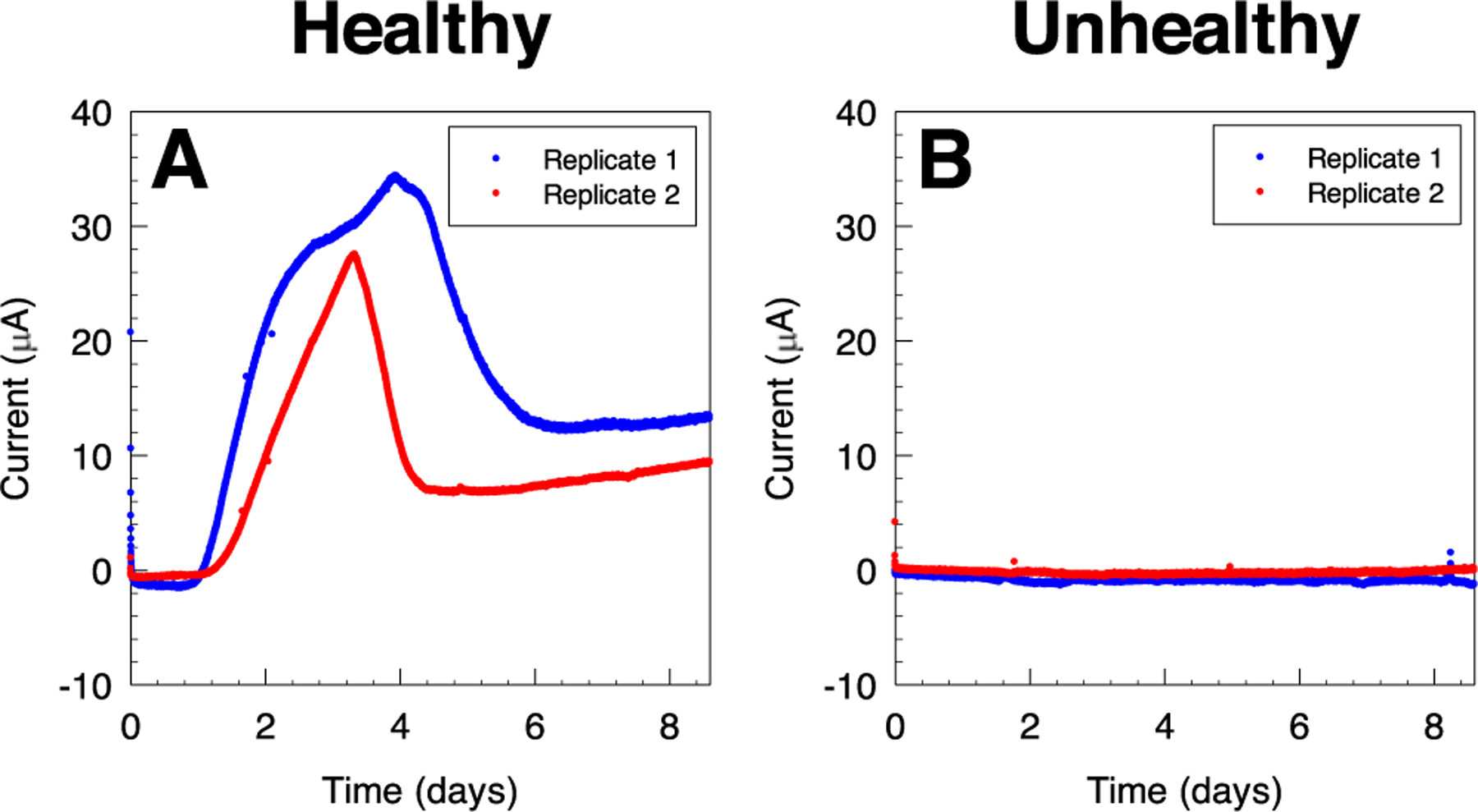
Chronoamperometric scans for electrodes deployed in (A) healthy and (B) unhealthy soil reactors. The electrodes were deployed 8 cm below the soil surface. Current measurements can be used to distinguish healthy from unhealthy soils.

Polarized electrodes were deployed in healthy and unhealthy soil reactors at 8 cm depth, and their potential was controlled at 0.3 V_Ag/AgCl_ (Fig. 3). Anodic current was observed within the first day of polarization in healthy soil reactors, and increased by 22.4 *μ*A and 10.5 *μ*A above baseline after 2 days in two biological replicates (Fig. 3A). Anodic current continued to increase, reaching a maximum of 34.4 *μ*A and 27.6 *μ*A after 3.9 days and 3.3 days of polarization, respectively. The measured current then decreased, reaching an average of 15.5 *μ*A and 11.8 *μ*A after 8 days. By comparison, no significant current change was observed in the electrodes deployed in unhealthy soil reactors (Fig. 3B). Anodic current in unhealthy soil reactors changed by −1.0 *μ*A and 0.0 *μ*A after 8 days in two biological replicates.

**Figure 4.**
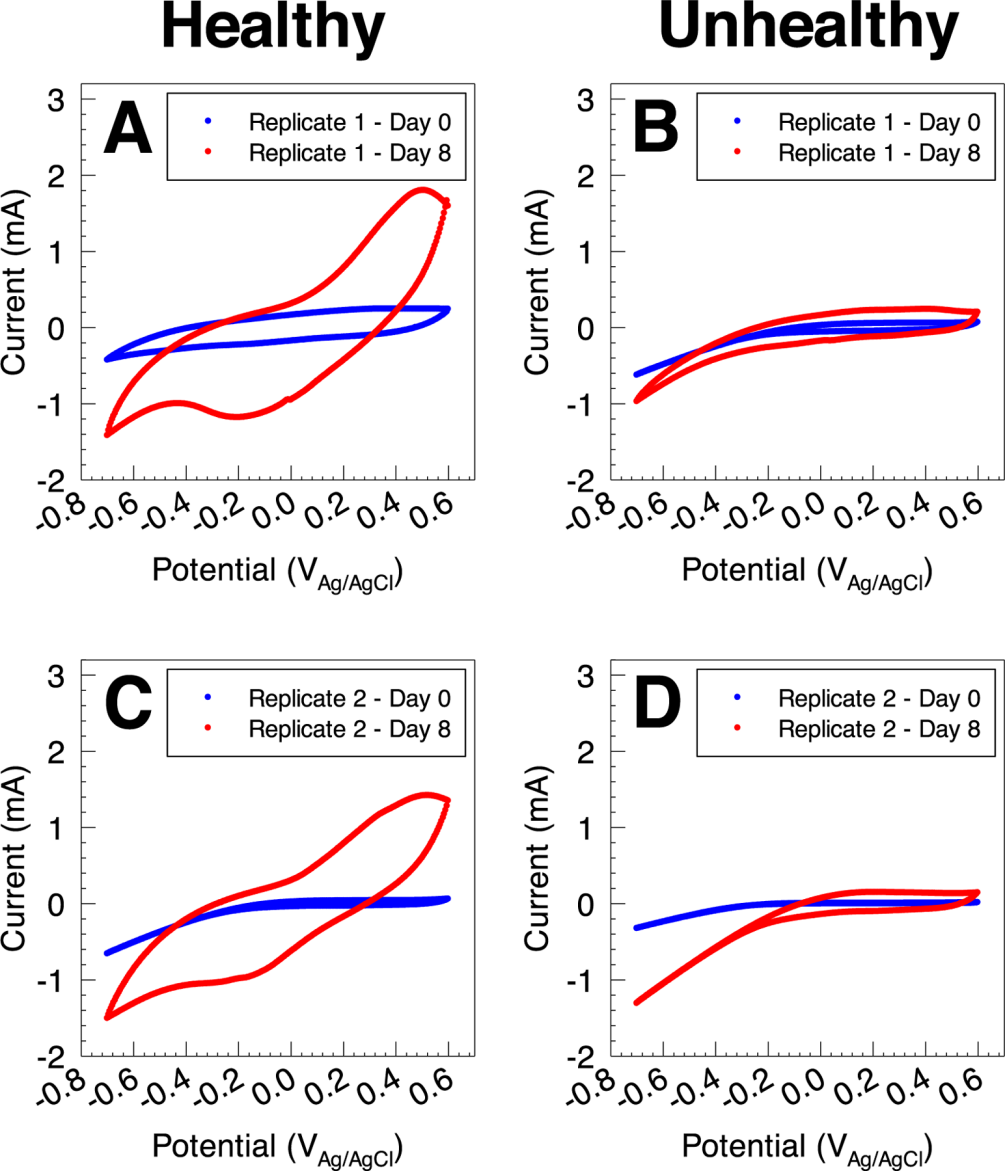
Cyclic voltammograms for electrodes deployed in healthy (A) and (C) and unhealthy (B) and (D) soil reactors. Cyclic voltammograms were recorded immediately after deployment (background) and after constant polarization for 8 days (after enrichment). Cyclic voltammograms indicate possible EAB biofilm enrichment on polarized electrodes in the healthy soil reactors.

We used CV to further investigate the electrochemical behavior of the polarized electrodes deployed in healthy and unhealthy soils. Cyclic voltammograms were recorded at two end points: 1) background cyclic voltammograms recorded immediately after assembling the soil reactors and prior to electrode polarization (day 0), and 2) after the enrichment of EABs recorded after constant polarization at 0.3 V_Ag/AgCl_ for 8.6 days (day 8). Background cyclic voltammograms (day 0) showed similar behavior in electrodes deployed in healthy and unhealthy soil reactors (Fig. 4). In both healthy and unhealthy systems, cyclic voltammograms consisted of a non-Faradaic background region and a cathodic wave below −0.2 V_Ag/AgCl_. Changes observed in the non-Faradaic region - mainly change in electrode capacitance - could be attributed to differences in the construction of the electrodes or differences in the soil structure in the vicinity of the electrode surface. The cathodic wave is likely due to abiotic oxygen reduction on the working electrode surface, which was previously documented to occur at a similar onset potential on the surface of carbon fabric electrodes.^32^ The difference in the magnitude of the reduction wave may be attributed to the variability of local oxygen concentration near the electrode surface in each reactor. Regardless, background cyclic voltammograms showed that all electrodes in both healthy and unhealthy soil reactors exhibited similar electrochemical behavior at time zero, with no evidence of electrochemical reactions coupled to biological metabolism or presence of EABs.

The electrochemical behavior observed in Cyclic voltammograms recorded after 8.6 days of constant polarization at 0.3V_Ag/AgCl_ provides evidence for anodic reactions, which are likely due to the enrichment of anodic EABs in healthy soil. An anodic behavior is observed above 0.1 V_Ag/AgCl_ in both electrodes deployed in healthy soil, with an anodic peak centered at 0.5 V_Ag/AgCl_ (Figs. 4A and 4C). This anodic reaction is likely the source of anodic current observed in constant polarization experiments in Fig. 3A, and indicates that enriched EABs utilize the polarized electrode as a terminal electron acceptor. Similarly, cyclic voltammograms of healthy soil reactors show a cathodic behavior below 0.1 V_Ag/AgCl_, with a cathodic peak observed at −0.17 V_Ag/AgCl_. In comparison, electrodes deployed in the unhealthy soil reactors exhibited similar behavior to the background cyclic voltammograms characterized by a non-Faradaic background region and a cathodic wave below −0.2 V_Ag/AgCl_ (Figs. 4B and 4D). Both replicates showed an increase in the capacitive current in the background region, which could be due to the adsorption of non-electrochemically active compounds from the soil onto the surface of the carbon electrode. Collectively, chronoamperometric scans and cyclic voltammograms show that anodic polarization selectively enhanced the electrochemical signals in electrodes deployed in healthy soil reactors.

**Figure 5.**
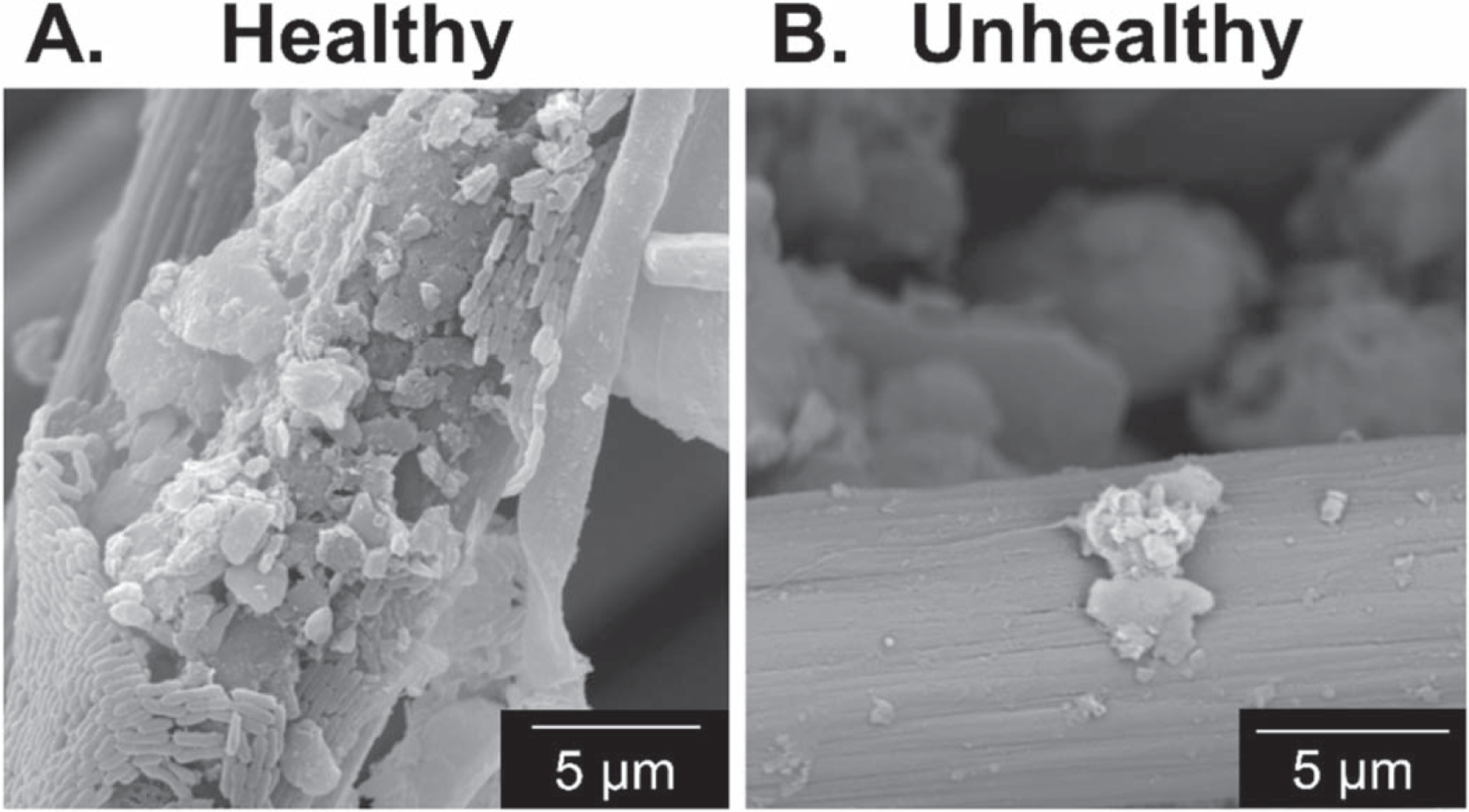
Representative SEM images of carbon fibers of the electrodes from healthy and unhealthy soils. In general, we observed the presence of microbes on the carbon fiber after polarization in healthy soil.

Healthy soils provide a suitable environment to support the metabolism and replication of soil microbes. We hypothesized that the electrochemical behavior observed in healthy soil could be due to the enrichment of EABs, where electrons generated through microbial metabolism could be transferred to the polarized electrode via extracellular electron transfer. Electrochemical data alone do not definitively show whether the observed electrochemical signals are a result of the enrichment of EABs or of specific redox reactions. Scanning electron microscopy (SEM) images provide secondary evidence to support the enrichment of EABs on the surface of polarized electrodes deployed in healthy soil reactors. Figure 5 shows representative images of carbon fibers of the carbon cloth electrodes harvested after polarization in healthy and unhealthy soils. Images of polarized electrodes in healthy soil reactors show the attachment of microbial populations around the carbon fiber strands. On the other hand, image of the electrodes from unhealthy soil reactors do not show the presence of microbial cells or attachment to the electrode surface. Put together, the electrochemical data and SEM images support that EABs could be enriched on carbon electrodes in soil systems, which could be monitored through electrochemical measurements. Because anodic current was observed only in healthy soil reactors, electrochemical signals can be used as an indicator for soil health.

### Response to the addition of glucose is observed in both healthy and unhealthy soil reactors.—

**Figure 6.**
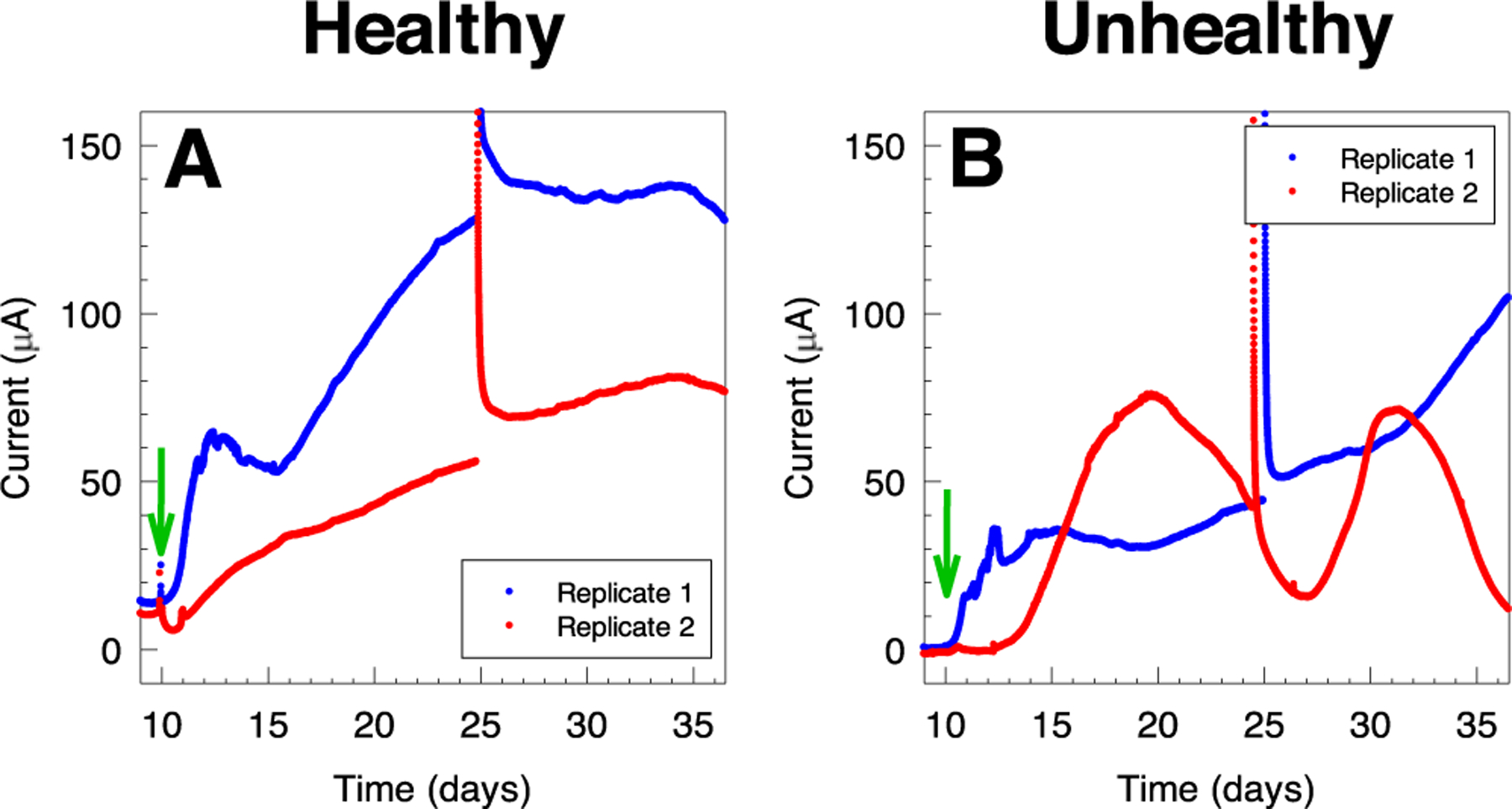
The response of polarized electrodes deployed in (A) healthy and (B) unhealthy soil reactors to the addition of glucose (green arrow). Anodic current increased in response to glucose addition in both healthy and unhealthy soils but showed variability. On the 25th day we disconnected the reactors and ran CV, which caused the discontinuity and jump in the data.

We attributed the selective observation of electrochemical signals due to the microbial colonization of electrode surface in the healthy soil reactors to the soil’s ability to support microbial metabolism and cell replication. To confirm this, we tested whether the soil amendment could stimulate the electrochemical signals in unhealthy soil reactors. Glucose addition is a common method in soil science to stimulate microbial activity in soil. For example, glucose addition was used to measure nitrogen fixation potentials^33^ and to stimulate soil respiration.^34^ A solution of glucose (both a carbon source and electron donor) was added to both healthy and unhealthy soil reactors that were polarized at 0.3 V_Ag/AgCl_ for 10 days. Figure 6 shows the current response following the amendment of soil reactors with glucose (time is indicated with a green arrow). Although healthy and unhealthy reactors started at a different baseline (13.8 *μ*A and 10.7 *μ*A for healthy soil compared to 0.4 *μ*A and −0.9 *μ*A for unhealthy soil), anodic current increased above baseline within 3 days after the addition of glucose. Healthy soil reactors reached a maximum of 138.2 *μ*A and 81.2 *μ*A, significantly higher than the maximum current observed prior to glucose addition. This indicated that the enrichment of EABs may be limited by the nutrient availability, even in healthy soil systems. Unhealthy soil reactors showed an increase in anodic current in both unhealthy soil replicates following glucose addition. However, both replicates showed a different temporal response. Replicate 1 showed an increase within the first day following glucose addition and reached a relatively steady response averaged at 32.6 *μ*A between 4 and 11 days after glucose addition. Afterward, the anodic current continued increasing and reached 110.1 *μ*A at the end of the experiment.

On the other hand, the anodic current in replicate 2 increased after 3 days of glucose addition, and reached a maximum of 76.0 *μ*A after 10 days. Afterward, the current dropped to a minimum of 15.3 *μ*A after 17 days, followed by an increase to reach a maximum of 71.4 *μ*A after 21 days, then continued to decrease until the end of the experiment. The cyclic behavior indicates instability in the anodic current generation in replicate 2, possibly due to factors causing self-inhibition within the enriched EAB. Nonetheless, anodic current was observed in all replicates in healthy and unhealthy soil reactors, which supports the hypothesis that the measured anodic current is a result of microbial metabolism. Additionally, the anodic current measured following glucose amendment was above the baseline observed in both healthy and unhealthy soils, suggesting that the metabolism of the enriched EABs was limited by nutrient availability in both soil systems.

**Figure 7.**
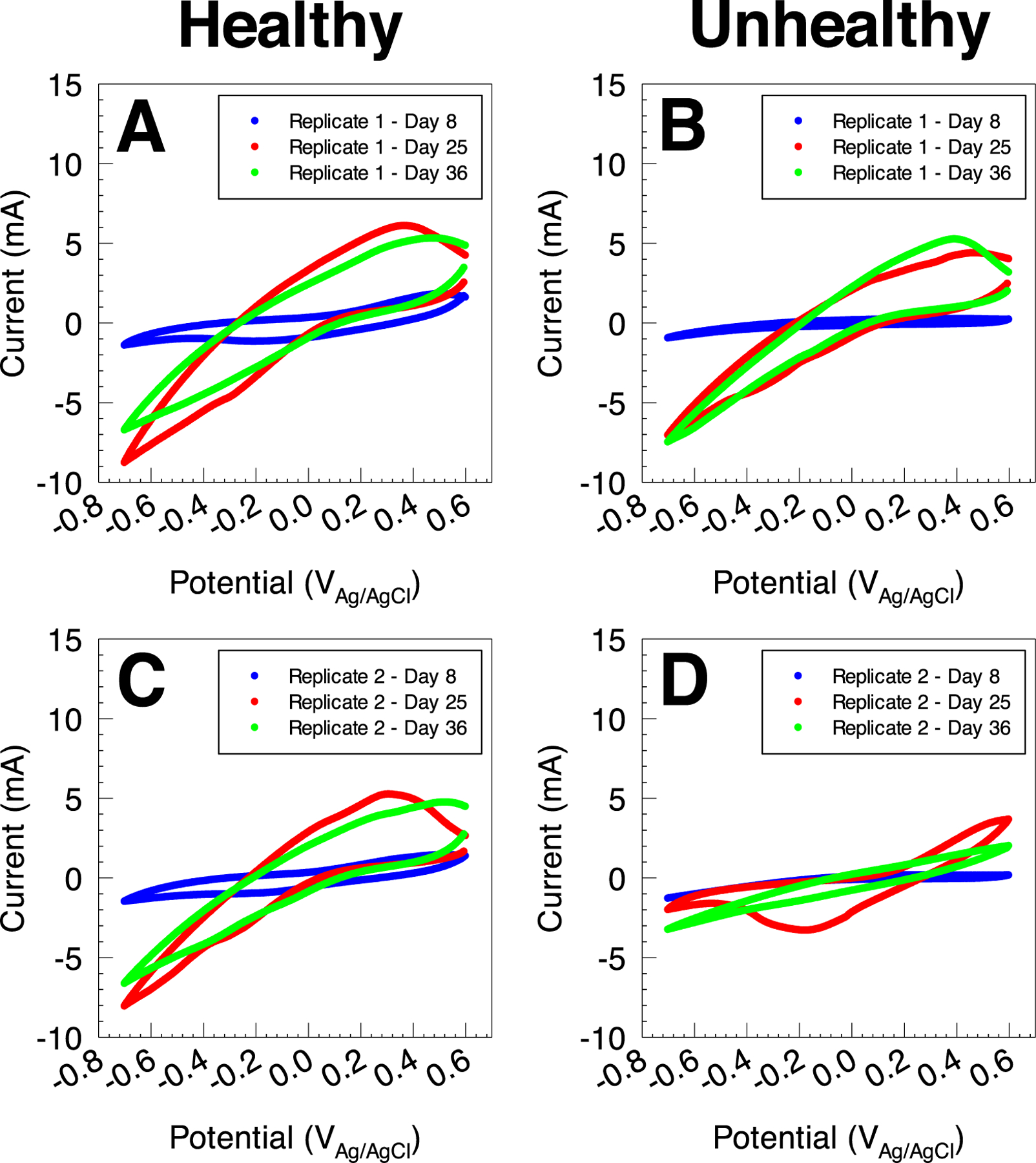
Cyclic voltammograms for electrodes deployed in healthy (A) and (C) and unhealthy (B) and (D) soil reactors. Cyclic voltammograms were recorded immediately after constant polarization for 8 days (day 8), and after the addition of glucose to soil and constant polarization for 17 and 28 days (day 25 and 36).

CV was used to compare the electrochemical behavior on the electrodes before and after glucose amendment (Fig. 7). Cyclic voltammograms were recorded 15 and 26 days after glucose amendment (day 25 and day 36 of the experiment). In both healthy and unhealthy soils, cyclic voltammograms recorded after glucose amendment showed a higher anodic current magnitude in comparison to cyclic voltammograms of enriched EABs prior to glucose amendment (Fig. 7A and 7C). In both replicates in healthy soil reactors, cyclic voltammograms recorded after 15 days of glucose amendment showed anodic region above −0.1 V_Ag/AgCl_, with an anodic peak centered at 0.3–0.35 V_Ag/AgCl_. Cyclic voltammograms recorded at days 36 show a similar anodic region with a comparable current magnitude while the anodic peak shifted to ∼0.5 V_Ag/AgCl_. Similar to the chronoamperometric data shown in Fig. 6B, the two replicates in unhealthy soil reactors showed a diverging response to glucose amendment (Fig. 7B and 7D). Replicate 1 showed a similar response to the healthy soil replicates, with an anodic region above −0.1 V_Ag/AgCl_ in cyclic voltammograms recorded at day 25 and day 36. Similarly, the anodic peak shifted from 0.4 V_Ag/AgCl_ at day 25 to 0.48 V_Ag/AgCl_ at day 36. On the other hand, cyclic voltammograms recorded at day 25 for replicate 2 showed an anodic region 0.2 V_Ag/AgCl_ which continued to increase with increasing applied potential; no anodic peak or mass-transport limited current region were observed. Interestingly, a defined cathodic region is observed below −0.2 V_Ag/AgCl_ for replicate 2 at day 25, with a defined cathodic peak centered at −0.17 V_Ag/AgCl_. Repeating CV recording at day 36 showed less defined anodic and cathodic regions, which is consistent with the current decrease from 46.7 *μ*A to 11.9 *μ*A observed in CA data between days 25 and 36. Overall, the electrochemical data shows that these signals can be connected to microbial metabolism of EAB attached to electrodes and that amending soils with glucose can lead previously distinct electrochemical patterns to converge to very similar patterns.

Monitoring electrochemical activities in soil samples is new, and research on this topic is limited. Recent literature mostly focused on monitoring electrochemical and redox activities in soil using inert electrodes. Our study extends the previous literature by comparing electrochemical activities in healthy and unhealthy soils. In this work, we compared the electrochemical signals using electrodes deployed in two soil samples (termed healthy and unhealthy) that were collected from sites used for wheat cultivation that showed differences in historic wheat grain yield. Future work will investigate the influence of different components of soil health (such as changes in soil organic matter content, physical properties and chemical composition of the soil and microbial community structure) on the observed electrochemical signals, and attempt to establish a functional relationship between electrochemical signals and the microbial communities present near the electrodes and in the soil. Additionally, future work will focus on transitioning electrochemical monitoring of soil health to the field. The experiments presented in this work were performed under defined laboratory conditions. In the field, the activity of microorganisms in soil is influenced by the daily and seasonal variations in temperature, light, substrate availability and plant metabolism. Transitioning our experiments to field conditions requires understanding the influence of these variations on the electrochemical signals measured in soil and requires the use of field-ready potentiostats that can be operated without access to the electrical power grid.^35,36^

## Conclusions

We explored the electrochemical signals using carbon cloth electrodes installed in healthy and unhealthy soils. We found that there is a redox gradient in both soils, with healthy soil showing prevalent reducing conditions compared to unhealthy soil. When electrodes were polarized at 0.3 V_Ag/AgCl_, anodic current was observed within a day of electrode polarization in healthy soils and while unhealthy soils remained at baseline levels. An increase in anodic activity was also observed in cyclic voltammograms of electrodes deployed in healthy soils following electrode polarization, while minimal change was observed in unhealthy soil electrodes. SEM images show the presence of microbes attached to electrodes in the healthy soil but not in the unhealthy soil. Glucose addition stimulated current in both types of soil and also caused differences in cyclic voltammograms between the two types of soil to converge. We conclude that healthy soil systems contain sufficient organic matter content and microbial diversity to establish reducing conditions in deeper parts of the soil and to support the growth of electrochemically-active biofilms. This could enable the use of electrical current measurement in the soil to distinguish between healthy and unhealthy soil.

## Supplementary Material

Supplementary Material

## List of Symbols

EPS: Extracellular polymeric substances
DOM: Dissolved organic matter
CV: Cyclic voltammograms
CA: Chronoamperometry
DPV: Differential pulse voltammetry
OCP: Open circuit potential
EAB: Electrochemically-active biofilm
SEM: Scanning electron microscopy
