# Supplementary material for: Electrochemically Active Biofilms as an Indicator of Soil Health
Source: J Electrochem Soc. Author manuscript; Available in PMC 2022 Oct 27. (PMC9608337; doi:10.1149/1945-7111/ac1e56)
Supplement: Supplementary Material [file NIHMS1835529-supplement-Supplementary_Material.pdf]

Supplemental Material for

Electrochemically active biofilms as an indicator of soil health

Abdelrhman Mohamed [1], Eduardo Sanchez [1], Natalie Sanchez [2], Maren L. Friesen [2,3] and Haluk Beyenal [1*]

[1] The Gene and Voiland School of Chemical Engineering and Bioengineering, Washington State University, Pullman, WA.
[2] Department of Plant Pathology, Washington State University, Pullman, WA
[3] Department of Crop and Soil Sciences, Washington State University, Pullman, WA

[*] Corresponding author:
Email: beyenal@wsu.edu ; Telephone: +1-509-335-6607 ; Fax: +1-509-335-4806

Table S1. LTAR data for the 'healthy' and 'unhealthy' soils used in this study. ID2: site reference number. Bulk Density: the weight of a soil column divided by its volume, TNConc: Total nitrogen concentration, TCConc: Total carbon concentration, TocConc: Total organic carbon concentration, TocStock: Total organic carbon stock (product of the TocConc and BulkDensity), TNStock: Total nitrogen stock (product of the TNConc and BulkDensity), pH: the pH of the soil.

Soil	ID2	Mean Relative Yield (1999 - 2015)	Latitude	Longitude	Top Depth (cm)	Bottom Depth (cm)	Bulk Density (g/cm^3)	TNConc (%)	TCConc (%)	TocConc (%)	TocStock (Mg/ha)	TNStock (Mg/ha)	pH
Healthy	333	1.069	46.782425	-117.080989	0	10	1.189	0.177	2.106	2.106	24.466	2.065	4.69
Unhealthy	195	0.963	46.780696	-117.078519	0	10	1.234	0.156	1.681	1.681	20.751	1.922	4.61

Table S2. Soil chemical analysis for the 'healthy' and 'unhealthy' soils used in this study.

	**Bulk Density**	**Bulk Density**	**Ec(1:1)**	**OM**	**NH_4^- N**	**NO_3^- N**	**Bray P1**	**Olsen P**	**Olsen K**	**SO_4^- S**	**Cl**	**B**	**Zn**	**Mn**	**Cu**	**Fe**
	g/ml	mill-lbs /ac-depth	dS/m	%	ppm	ppm	ppm	ppm	ppm	ppm	ppm	ppm	ppm	Ppm	ppm	ppm
**Healthy**																
H1	1.27	1.73	0.09	4.14	4.7	2.3	40	24	212	6	0.4	0.10	1.69	37.0	6.4	57
H2	1.3	1.77	0.08	4.16	3.9	1.6	37	20	177	5	0.5	0.07	0.33	34.4	1.1	58
H3	1.17	1.59	0.1	4.54	4.1	1.2	39	24	292	5	0.2	0.08	0.85	33.9	3.3	56
Mean	1.25	1.70	0.09	4.28	4.2	1.7	39	23	227	5	0.4	0.08	0.96	35.1	3.6	57
Standard deviation	0.0695	0.0945	0.01	0.225	0.42	0.56	1.5	2.3	58.9	0.6	0.2	0.02	0.69	1.66	2.7	1
**Unhealthy**																
U1	1.26	1.71	0.1	4.68	4.1	0.6	71	38	412	5	0.6	0.06	0.53	55.6	2	59
U2	1.26	1.71	0.09	4.68	3.7	0.8	67	36	316	4	0.4	0.04	1.63	53.4	5.9	53
U3	1.25	1.70	0.08	4.14	4.0	1.0	66	37	348	6	0.7	0.07	0.38	42.2	1.2	51
Mean	1.25	1.71	0.09	4.50	3.9	0.80	68	37	359	5	0.6	0.06	0.85	50.4	3.0	54
Standard deviation	0.0042	0.0058	0.01	0.312	0.21	0.20	2.6	1	48.9	1	0.2	0.02	0.68	7.19	2.5	4.2

	**Na**	**K**	**Ca**	**Mg**	**Total Bases**	**Base Saturation**	**pH (1:1)**	**A & E Buffer pH**	**Lime Req (to pH 6.5)**	**Al (KCl)**	**Al (DTPA)**
	meq/ 100g	meq/ 100g	meq/ 100g	meq/ 100g	meq/ 100g	%			lbs/ac	ppm	ppm
**Healthy**											
H1	0.03	1.05	7.8	2.1	11.0	70	5.08	7.16	3809	9	16
H2	0.02	0.81	8.0	1.9	10.7	68	4.90	7.1	4342	26	23
H3	0.03	0.89	6.5	1.8	9.2	68	5.00	7.13	4063	13	20
Mean	0.03	0.92	7.4	1.9	10.3	69	4.99	7.13	4072	16	20
Standard deviation	0.006	0.12	0.81	0.15	0.964	1.3	0.0902	0.0300	266.5	9	3.5
**Unhealthy**											
U1	0.05	0.54	8.6	2.7	11.9	69	4.85	7.13	4260	27	17
U2	0.03	0.45	7.7	2.3	10.5	68	4.85	7.15	4162	26	19
U3	0.04	0.75	7.8	2.6	11.2	70	5.06	7.22	3564	9	15
Mean	0.04	0.58	8.0	2.5	11.2	69	4.92	7.17	3995	21	17
Standard deviation	0.01	0.15	0.49	0.21	0.700	0.91	0.121	0.0473	376.5	10	2.0
